# An Inquiry into the Relative Efficiency of Water Filters in the Prevention of Infective Disease

**Published:** 1899-03

**Authors:** 


**An Inquiry into the Relative Efficiency of Water Filters in the Prevention of Infective Disease.** By G. Sims Woodhead, M.D., and G. E. Cartwright Wood, M.D. Pp. viii., 135. London: Printed at the Office of the British Medical Association. 1898.

This is a reprint in book form of a very valuable report upon all classes of domestic filters, with special reference to their power of removing pathogenic organisms from polluted water. A considerable number of filters, representing probably all the numerous types that have been placed upon the market, are examined in detail as regards their permeability both to ordinary water bacteria and to pathogenic forms. The authors arrive at a very important conclusion, already sufficiently recognised by those who have made a special study of the matter, but never perhaps formulated in so complete and authoritative a manner. None of the filters charged with the pulverulent or fibrous media, until lately so universally in use, afford the least protection against the passage of the causative agents of enteric fever or cholera; but, on the other hand, these filters are likely to prove sources of danger as secondary means of contaminating pure water. Differences in the rapidity of transmission of bacteria were noticed, and were attributed to the varying length of time necessary for washing the organisms through different thicknesses of filtering medium: and it would have added much to the interest of the report if the real efficacy of the sand filter-beds, upon which so much reliance is placed at the present time, had been tested on parallel lines. The only filters that were found to effectually exclude pathogenic bacteria were those in which the media employed were "forms of porcelain, compressed silicious earth and natural stone." In order, however, to bring about a sufficient rapidity of flow through these denser media, it is in general necessary to apply additional pressure, a proceeding which severely tests the mechanical fittings as well as the perfection of structure of the filter itself.

A second deduction from this noteworthy series of experiments is of great scientific interest; namely, that no filter is proof against bacteria capable of growth in the liquid undergoing filtration. No filter, therefore, permanently removes the ordinary water organisms which appear in the filtrate after a lapse of from two to four days, owing to growth along the pores of the filter. A precisely similar result is attained with typhoid and cholera bacilli if as little as one-fiftieth part of ordinary broth is added to the water; no such effect, however, is produced by the addition of sewage, water deposit, or other organic matters likely to occur in natural waters, and it seems improbable, therefore, that risk of this kind would be incurred in actual practice.

The whole report teems with exact and detailed information on many vexed questions; and will well repay perusal by all concerned in the maintenance of the public health.

